# Suicidal behaviors in the entertainment industry: a preliminary exploration of the interplay between work scheduling, social support, and wellbeing in Australia

**DOI:** 10.1186/s12888-022-04376-2

**Published:** 2022-12-01

**Authors:** Daniel Zarate, Christopher Sonn, Adrian Fisher, Vasileios Stavropoulos

**Affiliations:** 1 Institute for Health and Sport, Victoria University, Melbourne, Australia; 2 University of Athens, Athens, Greece

**Keywords:** Australian entertainment industry, Mental health, Multidimensional perceived social support, Suicide, Moderation analyses

## Abstract

**Objective:**

Workers of the Australian entertainment industry exhibit disproportionately high rates of impaired psychological wellbeing and suicidal behaviors, with such rates being exacerbated by the negative impact of working long and odd hours (Work Scheduling Impact; WSI). Nonetheless, stable and secure social support networks may buffer the risks associated with such systemic difficulties.

**Methods:**

The responses of 1302 Australian entertainment industry workers (50.3% females, M_age_ 38.39 years) on the Multidimensional Scale of Perceived Social Support, the Short Form Health Survey, WSI, and suicidal behaviors questions were examined via moderation analyses.

**Results:**

Higher social support and lower WSI appeared to reduce the suicidal ideation of those experiencing poorer mental health, while lower WSI further enhanced social support’s positive effect.

**Conclusions:**

Findings highlight the likely detrimental effect of WSI regarding the suicidal ideation reported by vulnerable Australian entertainment industry workers and stress the importance of the social support they experience.

**Public health implications:**

Interventions attempting to increase social support could improve inherent conditions associated with the Australian entertainment industry. Similarly, the negative effect of working long and odd hours on workers’ mental health and suicidal behaviors indicates the need to regulate the industry appropriately.

**Supplementary Information:**

The online version contains supplementary material available at 10.1186/s12888-022-04376-2.

## Introduction

Workers of the entertainment industry experience low rates of mental wellbeing and suicidal behaviors [1–3]. Difficulties associated with the nature of the entertainment industry, such as unstable employment and a ‘toxic culture’ have been proposed to underpin poorer mental wellbeing [2, 4]. For example, Van Rens and Heritage [3] reported that despite having high levels of resilience, circus performers showed higher depression, anxiety, and stress than 88%, 86%, and 77% of adults, respectively. Moreover, Van Den Eynde and colleagues [2] revealed that 44% of workers of the Australian entertainment industry have moderate to severe anxiety, 15% moderate to severe depression, and more importantly, four to five times greater incidence of suicide planning, resulting in more than double attempts compared to other Australians. Additionally, their work scheduling impact (WSI) due to working long and odd hours disrupts individuals’ social lives, could adversely affect their health, sleep [5], overall wellbeing [6–8], and likely increase their suicide risk. Thus, it is plausible that the WSI may associate with entertainment industry workers’ increased suicide behaviors and mental health issues.

In the present study, such suicide behaviors are considered to involve ideation, planning, and attempts [9–11]. Ideation is defined as the contemplations, wishes, and preoccupations with death and suicide; planning is the precursor of potentially lethal attempts; while attempts involve self-inflicted behaviors with non-fatal outcomes [12, 13]. Interestingly, factors related to suicidal behaviors may differently influence suicidal ideation, planning, or attempts over time [14]. For example, Joiner [15] illustrates that while mental disorders may result in higher suicide ideation, only certain disorders, such as posttraumatic stress disorder, may increase the risk for suicide attempts. Moreover, Klonsky and colleagues [14] suggest longitudinal variations of the same factors, such as loneliness and isolation, due to cumulative effects, with ideation being the initial step and planning and attempts following. To address such issues, the current study integrates four different measures concerning one’s suicidal behaviors, including ideation in the last 12 months and over one’s life, planning, and attempts. While assessing these distinct yet interrelated aspects, the present research considers self-reported social support as a potential protective condition [2, 16].

### Buffering effect of social support networks

Social support refers to one’s perception of social integration, bonds, and closeness to others in their surroundings [17]. Pearson [17] highlights three elements present in one’s sense of social support directly related to psychological wellbeing: (i) being cared for and loved, (ii) being valued, and (iii) experiencing a sense of belonging to a social group. Stable social support provides basic psychological and developmental needs such as belonging, self-esteem, and security with a direct (and positive) impact on depression, emotional stability, and suicidal behaviors [18]. Additionally, scholars proposed that social support has a buffering effect on burnout and work-related stress, as it may help individuals relax/distress from their perceived work demands [19]. Accordingly, one could assume that a strong social support network could buffer/moderate the risk of poor mental health and adverse WSI on suicidal behaviors among those working in entertainment.

### Current research

Systemic difficulties associated with the entertainment industry, such as WSI, have compromised workers’ wellbeing [2, 3]. Similarly, the buffering effect that social support systems have on daily stressors and poorer mental health has been demonstrated [19]. Despite this, there is limited research regarding the significance of the interplay between WSI (as a risk factor) and social support (as a potential buffer for suicidal behaviors) in workers of the Australian entertainment industry. Additionally, complex issues continue to affect the Australian entertainment industry (for example, a recent report revealed significant issues in the industry associated with toxicity, financial instability, and low mental wellbeing [20]), suggesting the need to explore these issues further.

**Fig. 1:**
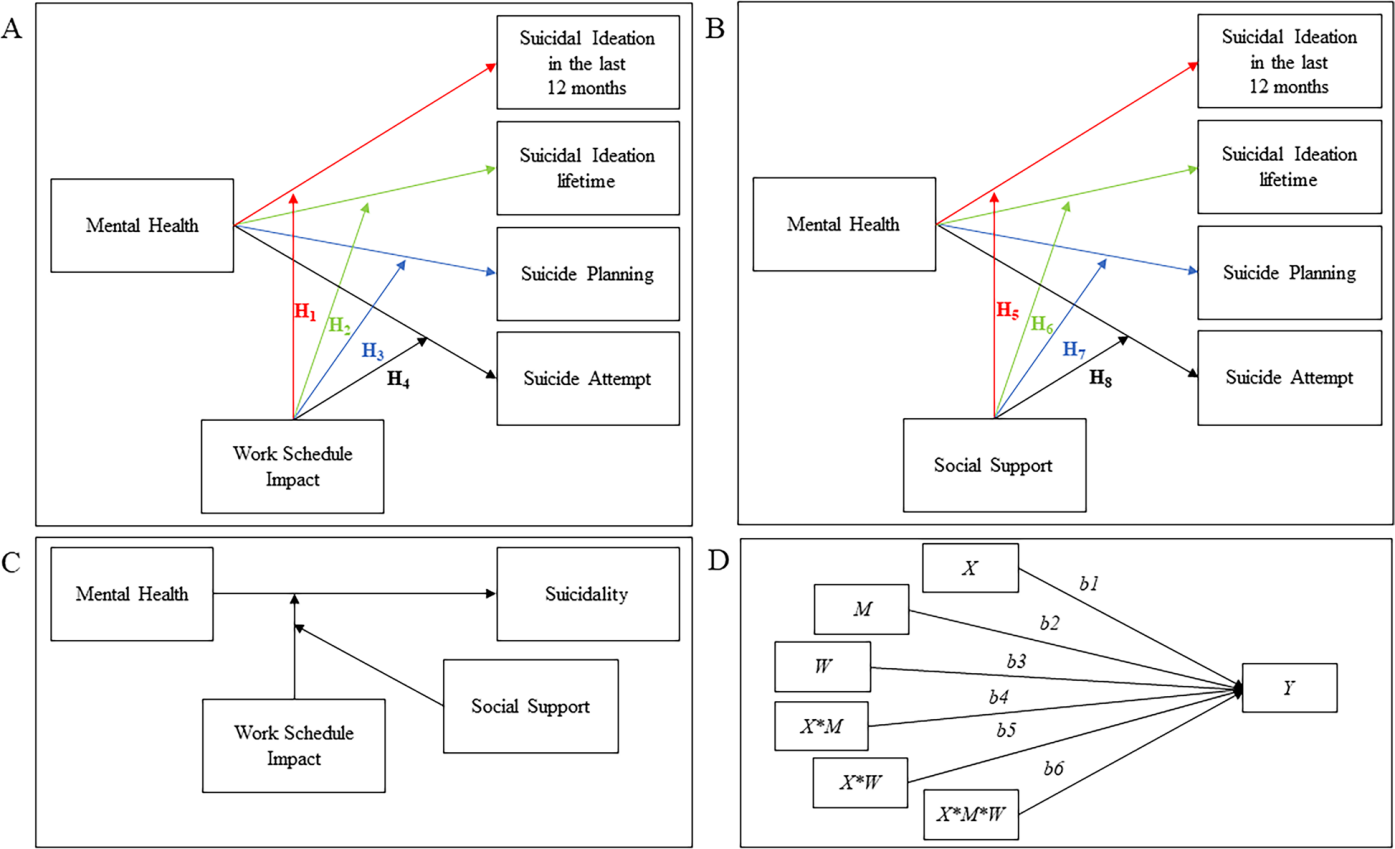
This figure shows the hypothesised relationships between selected variables. Panel **A** presents a conceptual diagram of H_1_*,* H_2_, H_3_, H_4._ Panel **B** on the right illustrates a conceptual diagram of H_5_*,* H_6_, H_7_, H_8._ Panel **C** (bottom left) presents a conceptual diagram of H_9_ (suicide ideation in the last 12 months)*,* H_10_ (suicide ideation lifetime), H_11_ (suicide planning), H_12_ (suicide attempts)_._ Finally, panel **D** (bottom right) shows a conceptual diagram of moderated moderation effects illustrating that this model aims to test (i) the moderating effect of M on the relationship between X and Y (*b*_*4*_), (ii) the moderating effect of W on the relationship between X and Y (*b*_*5*_), and (iii) the concurrent effect of both moderators M and W on the relationship between X and Y (*b*_*6*_)

The present study aims to address this gap by emphasizing how the WSI-Social support interaction could associate with the likelihood of Australian, entertainment industry workers experiencing lower wellbeing, to engage in suicidal behaviors. Specifically, it combines unique conceptual and methodological strengths to translate its findings into specific practical implications. Findings aspire to provide vital information to policymakers via: (1) shedding light on the need, and the directions, of regulating the precarious position of those working in the entertainment industry to potentially regulate their WSI; and (2) demonstrating the importance of social support networks in suicide prevention. To address these aims, the following hypotheses were formulated:

- Workers experiencing lower mental health/wellbeing and higher WSI will report higher suicidal ideation in the last 12 months (H_1_), over their lifetime (H_2_), suicide planning (H_3_), and suicide attempts (H_4_, Fig. 1 panel A) compared to those showing lower WSI.
- Workers experiencing lower mental health/wellbeing and higher levels of social support will report lower suicidal ideation in the last 12 months (H_5_), suicidal ideation in participants’ lifetime (H_6_), suicide planning (H_7_), and suicide attempts (H_8_, Fig. 1 panel B) compared to those showing lower social support.
- Workers experiencing higher levels of social support will report lower suicidal ideation in the last 12 months (H_9_), suicidal ideation in participants’ lifetime (H_10_), suicide planning (H_11_), and suicide attempts (H_12,_ Fig. 1 panel C and D), compared to their counterparts experiencing concurrently the same level of mental health/wellbeing and WSI adversity.

## Method

### Participants

A total of 2409 workers of the Australian entertainment industry completed an online survey for a project titled “Working in the Australian Entertainment Industry: Final Report” between 23/01/2015 and 08/05/2015 [2]. The original survey included a series of questionnaires beyond the scope of the current study. A filter question/item related to one’s experience of suicidal thoughts and/or actions, which constitutes the specific interest of the present study, was positively answered by 1302 respondents, who additionally addressed the suicide-specific measures analyzed here. Thus, the current study used a sample of 1302 participants (*M*_*age*_ = 38.39, *SD*_*age*_ = 0.98; 50.3% females). Missing responses (*n* = 331, 6.1%) were completely at random (MCAR *Little’s test: χ*^*2*^_[1096]_ = 0.065), and thus a listwise deletion was conducted. A-priori power analyses via the G-power software [21] indicated that for two tails of significance, F^2^ = 0.15, α error probability = 0.05, Power (1- β error probability) = 0.95 and seven predictors (i.e., wellbeing/mental health, WSI, social support, wellbeing/mental health x WSI, wellbeing/mental health x social support, social support x WSI, wellbeing/mental health x social support x WSI), a total sample of 89 was required for an actual power of 0.95, at a critical *t* = 1.989, *df* = 81. Workers of this industry belonged to several employment categories ranging from musicians, circus performers, dancers, managers, directors, and sound technicians. Supplementary Table 1 presents participants’ key demographic statistics.

### Measures

The *Multidimensional Scale of Perceived Social Support (MSPSS)* was employed to measure subjective perceived social support [22]. A total of 12 items loads on three subscales (four per subscale, i.e., friends, family, and significant other) rated on a seven point-Likert scale (1 = *very strongly disagree* to 7 = *very strongly agree*) with lower scores indicating lower perceived support. Examples of items include *“There is a special person who is around when I am in need”*. Total item responses were added to reflect one’s social support (as a whole and per dimension), ranging between 12–84, and 4–48, respectively. The internal consistency for one’s overall social support in the present study was satisfactory (Cronbach’s *α* = 0.91, McDonald’s ω = 0.91, see Supplementary Table 2 [23]).

The *Short Form 12 items Health Survey (SF12)* was used to assess mental health [24]. A total of 12 items loads on two subscales (i.e., mental, and physical component) and include items such as *“As a result of emotional problems, have you accomplished less than you would like?”*. Standardised scores suggest a mean of 50 (*SD* = 10) with lower scores indicating lower mental wellbeing. The internal consistency for the present study was satisfactory (*α* = 0.88; ω = 0.88; Supplementary Table 2). Considering the focus of the study, we only employed the mental health component (MHC) of the scale.

#### Work Schedule Impact (WSI)

The scale determines the impact on wellbeing that a particular work schedule has [2]. The scale consists of five items loading on two subscales (intra-individual and inter-individual impact) and includes items such as *“I have trouble maintaining my social life as a result of my work schedule”*. Items were rated on a five point-Likert scale with higher scores indicating a higher impact due to work schedules. The questionnaire exhibited internal consistency (*α* = 0.81; ω = 0.81; see Supplementary Table 2) and appropriate factorizable properties (EFA; KMO = 0.76, Bartlett’s χ^2^ [15] = 2861; *p* < 0.001), item loading 0.50 to 0.92; see Supplementary Table 3).

#### Suicidal behaviors

Following previous suggestions [2, 9], specific questions with dichotomous outcomes (yes/no) were employed to assess all three aspects of suicidal behaviors (i.e., ideation, planning, and attempts). Three questions were employed to assess participants’ suicide ideation in their lifetime (i.e., *“In your lifetime, have you ever felt that life was not worth living?*) and three questions to assess participants’ suicide ideation in the last 12 months (i.e., *“In the last 12 months, have you ever thought of taking your life, even if you would not really do it?”*). Scores were added for suicidal ideation in the last 12 months and lifetime, awarding one point per each ‘yes’ with a score range of 0–3 (high scores represent high suicidal ideation). These questions showed excellent internal consistency (*α* = 0.87; ω = 0.87; see Supplementary Table 2). Similarly, the question *“Have you planned to complete suicide?”* was employed to assess participants’ intention to act on suicidal thoughts without necessarily involving preparatory behaviors to die by suicide. Finally, the question *“Have you ever made an attempt to take your life?”* was employed to assess participants’ suicide attempts.

### Procedure

A Victoria University research team, in conjunction with Entertainment Assist, conducted a project titled ‘Working in the Australian Entertainment Industry: Final Report’ attempting to identify health and wellbeing concerns for those who work in this industry [2] was approved by the Victoria University Ethics Committee (HRE14-270). Eligible participants, adult workers of the Australian creative industry, were invited to complete an online survey advertised through Entertainment Assist’s membership email list and their Facebook site. Before completing the survey, participants accessed information about the voluntary and anonymous nature of the study, its aims, significance, and their right to withdraw at any point and without repercussions via the Plain Language Information Statement.

### Statistical analysis

A series of regression-based moderation analyses were conducted on IBM-SPSS 26 using the Process macro [25]. Specifically, this study used a model with two moderators (WSI and MSPSS) aiming to assess (i) the rate of change in the relationship between mental health (SF12) and suicidal behaviors at different levels of these moderators and (ii) the concurrent associations of both moderators. The moderating association of WSI on the relationship between mental health and suicidal behaviors was assessed by the interaction SF12*WSI, and the moderating association of MSPSS on the relationship between mental health and suicidal behaviors was assessed by the interaction WSI*MSPSS. Finally, a higher order interaction between the independent variable and both moderators (SF12*WSI*MSPSS) was used to observe the concurrent association of the interplay between work schedule and social support on the relationship between mental health and suicidal behaviors (for visual clarification see Fig. 1 panel C and D). Following suggestions outlined in Hayes [25], mean centring was conducted on variables that defined products, and simple slope analysis was used to visualize conditioning values at the 16^th^, 50^th^, and 84^th^ percentiles. Subsequently, the Johnson-Neyman technique was used to provide more detail in visualizing moderating effects [25].

Several models were employed to test the hypothesized relationships. To test H_1_, H_5_, and H_9_, SF12 was used as the independent variable, WSI, MSPSS and their interactions were the moderators, and suicidal ideation in the last 12 months the outcome variable. The same model/structure was then reapplied with different outcome variables: (a) the suicidal ideation in participants’ lifetime as the outcome was employed to address H_2_, H_6_, and H_10_; (b) with suicide planning as the outcome regarding H_3_, H_7_, and H_11;_ and (c) suicide attempts as the outcome variable considering H_4_, H_8_, and H_12_. Due to the last two models referring to binary outcomes, logistic regression instead of linear was calculated, while bootstrapping at 1000 resamples was applied for all analyses.

## Results

To assess the moderating association of the interplay between work scheduling (WSI) and social support (MSPSS) on the relationship between mental wellbeing and suicidal ideation over the last 12 months (hypotheses H_1_, H_5_, and H_9_), SF12 was employed as the independent variable, WSI as the moderator, MSPSS as the moderating moderator, and suicidal ideation in the last 12 months as the outcome variable. The model explained 27.3% of the variance in suicidal ideation during participants’ last 12 months (*R*^2^ = 0.2729, *F*_(7, 1294)_ = 69.380, *p* < 0.001), with SF12 (*b*_11_ = -0.042, *p* < 0.001), WSI (*b*_12_ = 0.034, *p* < 0.001), and MSPSS (*b*_13_ = -0.372, *p* < 0.001) as significant predictors.

**Fig. 2:**
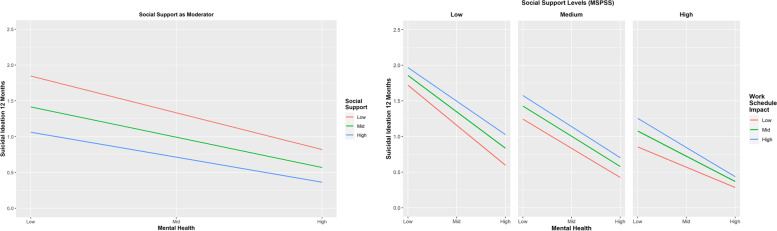
The left panel shows the significantly moderating effect of social support (MSPSS) on the relationship between mental health (SF12) and suicide ideation in the last 12 months. The right panel shows the combined (and significant) moderating effect of work schedule impact (WSI) and social support (MPSS) on the relationship between mental health and suicide ideation in the last 12 months

Considering H_1_, the moderating role of WSI on the relationship between SF12 and suicidal ideation in the last 12 months (*b*_14_ = -0.001, *p* = 0.724) was not significant. Considering H_5_, the moderating role of MSPSS on the relationship between SF12 and suicidal ideation in the last 12 months (*b*_15_ = 0.010, *p* = 0.008) was significant. As predicted, when MSPSS increased by one unit, the coefficient for the moderating effect of SF12 on suicidal ideation in the last 12 months increased by 0.01 (Table 1 and Fig. 2-left panel). Finally, considering H_9_, the three-way interaction SF*WSI*MSPSS was significant (*b*_16_ = -0.001, *p* = 0.018) explaining 0.32% of the variance. When MSPSS increased by one unit, the coefficient for the moderating effect of SF12 and WSI on suicidal ideation in the last 12 months decreased by 0.001 (see Fig. 2, right panel for low, medium and high levels of MSPSS). Interestingly, the interaction between SF12 and WSI only became significant at MSPSS 85^th^ percentile (MSPSS > 9.78; see Supplementary Table 4). In other words, the exacerbating role of WSI on the relationship between mental health and suicide ideation in the last 12 months was significantly mitigated at high levels of perceived social support.

**Table 1:** Estimating regression coefficients with ‘Suicidal ideation in the last 12 months’ and ‘Suicidal ideation in participants lifetime’ as outcome variables

Variable	***b***	**BSE**	***t***	***p***	**BLLCI**	**BULCI**
Outcome Variable—Suicidal Ideation in the last 12 months						
*a*: Constant/Intercept	1.0023	.0347	30.0849	.0001	.9325	1.0686
*b*_*11*_: SF12	-.0418	.0033	-12.4114	**.0001**	-.0484	-.0356
*b*_*12*_: WSI	.0341	.0078	4.5814	**.0001**	.0186	.0494
*b*_*13*_: MSPSS	-.3720	.0420	-8.6329	**.0001**	-.4530	-.2873
*b*_*14*_: SF12*WSI	-.0002	.0005	-.3528	.7243	-.0013	.0008
*b*_*15*_: SF12*MSPSS	.0103	.0033	2.6408	**.0084**	.0037	.0167
*b*_*16*_: SF12*WSI*MSPSS	-.0014	.0004	-2.3729	**.0178**	-.0022	-.0006
Outcome Variable—Suicidal Ideation in their lifetime						
*a*: Constant/Intercept	1.9618	.0351	57.3726	.0001	1.8930	2.0311
*b*_*21*_: SF12	-.0253	.0035	-7.3337	**.0001**	-.0321	-.0185
*b*_*22*_: WSI	.0295	.0079	3.8673	**.0001**	.0134	.0447
*b*_*23*_: MSPSS	-.3052	.0448	-6.9498	**.0001**	-.3923	-.2172
*b*_*24*_: SF12*WSI	.0019	.0006	3.0794	**.0020**	.0007	.0030
*b*_*25*_: SF12*MSPSS	-.0036	.0037	-.8903	.3735	-.0109	.0036
*b*_*26*_: SF12*WSI*MSPSS	-.0002	.0005	-.4089	.6827	-.0011	.0007

*b* Bootstrapped estimated value of unstandardised regression coefficient, *BSE* Bootstrapped standard error, *t* T-test statistic, *p* Probability, *BLLCI* Bootstrapped lower-level confidence interval, *BULCI* Bootstrapped upper-level confidence interval, *SF12* Short Form 12 items Mental Health Component, *WSI* Work Schedule Impact, *MSPSS* Multidimensional Scale of Perceived Social Support

To assess the moderating effects of role of the interplay between work scheduling (WSI) and social support (MSPSS) on the relationship between mental wellbeing and participants’ suicide ideation over their lifetime (hypotheses H_2_, H_6_, and H_10_), WSI was used as moderator, MSPSS as moderating moderator, and suicidal ideation in participants’ lifetime as the outcome. The model explained 18.3% of the variance (*R*^2^ = 0.1824, *F*_(7, 1294)_ = 41.251, *p* < 0.001), with SF12 (*b*_*21*_ = -0.025, *p* < 0.001), WSI (*b*_*22*_ = 0.029, *p* < 0.001), and MSPSS (*b*_*23*_ = -0.305, *p* < 0.001) being significantly associated to suicidal ideation in a participants’ lifetime.

**Fig. 3:**
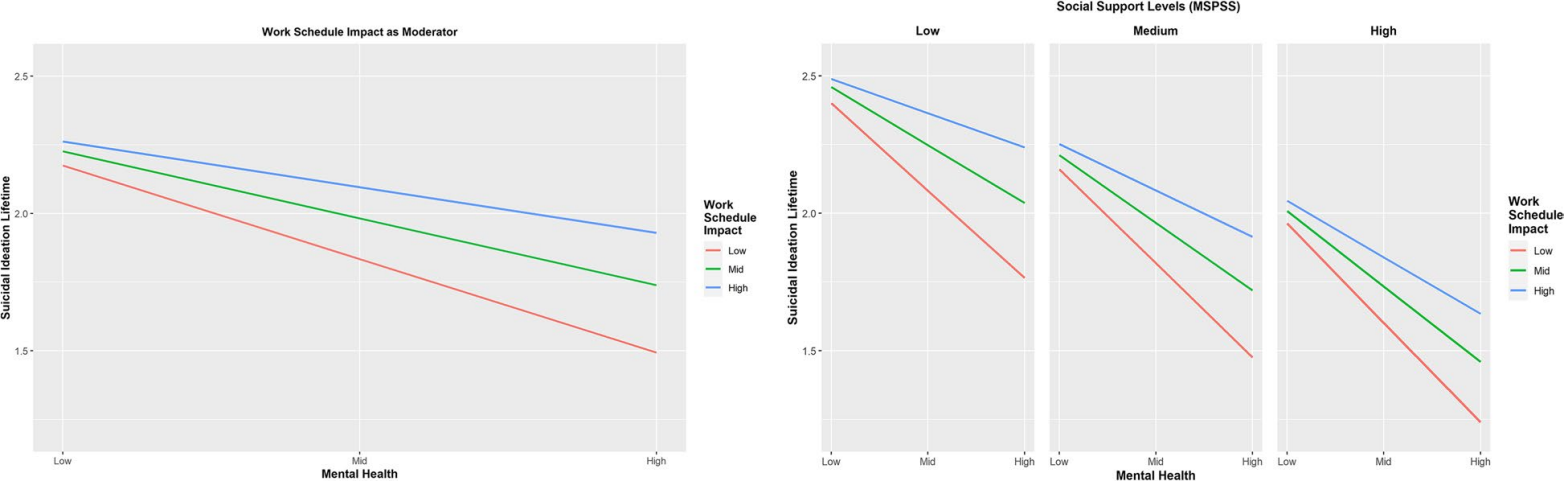
The left panel shows the moderating effect of work schedule impact (WSI) on the relationship between mental health (SF12) and suicide ideation in participants’ lifetime. The right panel shows the combined moderating effect of work schedule impact (WSI) and social support (MPSS) on the relationship between mental health (SF12) and suicide ideation in participants’ lifetime

Considering H_2_, the moderating role of WSI on the relationship between SF12 and suicide ideation in participants’ lifetime (*b*_24_ = 0.002, *p* = 0.002) was significant. When WSI increased by one unit, the relationship between SF12 and suicidal ideation in participants’ lifetime increased by 0.002 (Table 1 and Fig. 3, left panel). Considering H_6_ and H_10_, the moderating role of MSPSS on the relationship between SF12 and suicidal ideation in the last 12 months (*b*_25_ = -0.004, *p* = 0.373) and the three-way interaction SF*WSI*MSPSS (*b*_26_ = -0.001, *p* = 0.683) were not significant (see Fig. 3, right panel regarding low, medium and high MSPSS).

To assess the moderating role of the interplay between work scheduling (WSI) and social support (MSPSS) on the relationship between mental wellbeing and suicide planning (hypotheses H_3_, H_7_, H_11_), a logistic regression model with SF12, WSI, and MSPSS as independent variables and suicide planning as the outcome variable was used. Interaction terms between independent variables were included in the model to assess potential moderating associations. The model represented a significant improvement with respect to a null model (*χ*^2^_[6] _= 77.149, *p* < 0.001) and correctly classified 70.3% of cases. The Nagelkerke pseudo *R*^2^ indicated that the predictors accounted for approximately 8.4% of the variance in suicide planning. However, no single predictor or interaction term significantly predicted suicide planning (Table 2). Given that Wald’s coefficient was largest for SF12*WSI (*b*_*34*_ = 0.340) and SF12*MSPSS (*b*_*35*_ = 0.370), it is likely that the interaction terms account for most of the overlapping variance otherwise associated with single predictors.

**Table 2:** Logistic regression coefficients with ‘Suicide planning’ and ‘Suicide attempts’ as outcome variables

						**95% BCI for Exp(B)**	
Variable	*B*	BSE	Wald	*p*	Exp(B)	Lower	Upper
Outcome Variable—Suicide planning							
*a*: Constant/Intercept	.735	1.840	.160	.689	2.086		
*b*_*31*_: SF12	-.032	.065	.244	.622	.969	.853	1.099
*b*_*32*_: WSI	-.013	.058	.047	.828	.987	.881	1.107
*b*_*33*_: MSPSS	-.106	.307	.118	.731	.900	.493	1.643
*b*_*34*_: SF12*WSI	.002	.003	.340	.560	1.002	.996	1.007
*b*_*35*_: SF12*MSPSS	-.008	.013	.370	.543	.992	.967	1.018
*b*_*36*_: SF12*WSI*MSPSS	.000	.001	.000	.999	1.000	.999	1.001
Outcome Variable—Suicide attempts							
*a*: Constant/Intercept	-.931	2.358	.156	.693	.394		
*b*_*41*_: SF12	-.044	.086	.258	.611	.957	.808	1.133
*b*_*42*_: WSI	.030	.074	.162	.688	1.030	.891	1.192
*b*_*43*_: MSPSS	-.200	.390	.264	.608	.819	.381	1.757
*b*_*44*_: SF12*WSI	.002	.004	.197	.657	1.002	.995	1.009
*b*_*45*_: SF12*MSPSS	.004	.017	.063	.801	1.004	.971	1.038
*b*_*46*_: SF12*WSI*MSPSS	.000	.001	.305	.581	1.000	.998	1.001

*B* Logistic coefficient, *BSE* Bootstrapped standard error, *p* Probability, *Exp(B)* Odds ratio, *BCI* Bootstrapped confidence interval, *SF12* Short Form 12 items Mental Health Component, *WSI* Work Schedule Impact, *MSPSS* Multidimensional Scale of Perceived Social Support

Finally, to assess the moderating role of the interplay between work scheduling (WSI) and social support (MSPSS) on the relationship between mental wellbeing and suicide attempts (hypotheses H_4_, H_8_, H_12_), a logistic regression model was run with SF12, WSI, and MSPSS as independent variables and suicide attempts as the outcome variable. Interaction between independent variables was also included in the model to assess potential moderating effects. The model represented a significant improvement with respect to a null model (*χ*^2^_[6] _= 38.331, *p* < 0.001) and correctly classified 86.5% of cases. The Nagelkerke pseudo *R*^2^ indicated that the predictors accounted for approximately 5.5% of the variance. However, as with suicide planning, no single variable or interaction term was significantly associated with suicide planning (Table 2). In this case, larger Wald coefficients were observed for the three-way interaction SF12*WSI*MSPSS (*b*_*46*_ = 0.305), MSPSS (*b*_*43*_ = 0.264), and SF12 (*b*_*41*_ = 0.258), suggesting their comparative importance.

## Discussion

### Moderating effect of work scheduling impact (WSI) on the relationship between mental health and suicidal behaviors

This study has been (to the best of the authors’ knowledge) the first to distinctly evaluate the buffering association of social support networks, as well as the exacerbating association of work scheduling impact (WSI) with suicidal ideation, planning, and attempts of workers in the Australian entertainment industry. Findings indicated that mental health, WSI, and social support were significantly associated with workers’ suicide ideation in their lifetime (H_2_). More importantly, high impact due to work scheduling (WSI) was shown to significantly associate with an increase in suicide ideation even in workers with good mental health. This highlights the disruptive association between working long and odd hours and suicidal behaviors among Australian entertainment workers, denoting the need to regulate labor conditions in their industry appropriately [2, 7].

Alternatively, the moderating effect of WSI was not present in workers’ suicide ideation in their last 12 months (H_1_), suicide planning (H_3_), and suicide attempts (H_4_). This denotes that (i) a more comprehensive approach to capture variables influencing suicide planning and attempts may be needed to increase our understanding of such behaviors, and (ii) that the exacerbating role of WSI on suicide ideation may not always be present during the initial 12 months of experiencing problems, due to working long and odd hours. In line with previous literature [26], this could imply that work-related stress may have a progressive or accumulated effect on one’s mental health and suicide ideation, which could become more tangible after 12 months of experiencing impaired sleep and disrupted social life associated to one’s work commitments.

### Moderating effect of social support on the relationship between mental health and suicidal behaviors

Considering the buffering effect of social support on the relationship between mental health and suicidal behaviors, results provide mixed support to the proposed hypotheses. Specifically, support was only observed for our hypothesized association between lower social support and the relationship between mental health and suicide ideation in the last 12 months (H_5_). This association is particularly important for workers of the Australian entertainment industry who may experience low social support considering their higher reported suicide ideation levels (Fig. 2 left panel). This is in line with past literature suggesting that increased levels of social support are associated with belongingness, social integration, feeling supported, and consequently improved mental wellbeing [2, 16, 19].

The buffering association of social support was not revealed considering suicide ideation lifetime (H_6_), suicide planning (H_7_), and suicide attempts (H_8_). The lack of support for these hypotheses may indicate the potential importance of other individual and situational factors related to one’s suicide behaviors beyond the level of social support experienced. Thus, it may be suggested that a ‘systems approach’ incorporating a range of protective and risk factors, at both the individual and the contextual level, as well as their interactions, should be considered to assess the transitioning from ideation to planning or execution and suicide behaviors in general [11]. Additionally, Klonsky and May [14] suggest that a progressive transition from moderate to severe suicide ideation usually occurs when hopelessness is greater than a sense of connectedness. This could explain the buffering association of social support on suicide ideation *only* during the first 12 months.

### Moderated moderation – effect of WSI and social support on the relationship between mental health and suicidal behaviors

Finally, mixed results were observed considering the proposed interplay (moderated moderation effects) between WSI and social support regarding the relationship between mental health and suicidal behaviors among Australian entertainment industry workers. Specifically, our hypothesized role of the interaction between WSI and social support on the association between one’s level of reported mental health and suicide ideation in the last 12 months was confirmed (H_9_). Thus, one could suggest that while the nature of the entertainment industry may involve doing shift work (e.g., live performances are likely to happen at night or during the weekend), appropriately regulating the maximum number of working hours per day, providing fair remuneration (e.g., recognition of over-time) and compensation for working odd hours (e.g., time in lieu) may likely associate to weakening suicidal behaviors among more vulnerable workers. Indeed, past research identified *financial instability* as one of the main concerns associated with working in the Australian entertainment industry, signalling a power dynamic that may prevent workers from declining opportunities for paid work having to endure long and odd hours despite potentially adverse psychological consequences [2]. These recommendations seem particularly relevant considering that the effects of the Covid-19 pandemic may be largely unexplored, particularly in populations whose financial security has been severely impacted due to lengthy periods of social restrictions.

Finally, it is important to denote the lack of support for proposed moderating effects associated with participants’ suicide planning and attempts. This likely indicates that while both WSI and social networks are significantly associated with suicide behaviors, other personal traits/experiences are required for one to transition from suicidal ideation to planning and action. Indeed, Overholser and Spirito [27] propose that only a small proportion of individuals thinking about suicide attempt to take their life, with impulsivity and hopelessness due to the inability to improve situational contexts playing an important role. This highlights the importance of appropriately addressing differences in the assessment and prevention of suicide ideation, planning, and attempts [11]. Thus, additional cautiousness is recommended in the interpretation of the current findings.

### Limitations and future research

Despite the value of these findings and the methodological strengths of the current study, limitations need to be considered. Firstly, given the cross-sectional nature of the employed data, collected in 2015, causative associations could only be speculated here, while potential changes during later developments may have not been accounted. Secondly, considering the inherent cultural elements of different countries, it is important to note that the findings presented in the current research might not be generalizable to workers of the entertainment industry in other parts of the world, and perhaps future research may seek to employ statistical methods to validate these results (i.e., measurement invariance [28, 29]). Thirdly, only self-report measures were used, increasing the risk of subjectivity biases. Hence, longitudinal studies assessing variables of relevance in workers of the entertainment industry of different countries while concurrently employing more objective measures (i.e., one’s actual work schedule to assess WSI) would be recommended. Fourthly, considering that a time criterion was only employed to assess suicide ideation (i.e., 12 months vs. lifetime), a potential confounding effect of recent/lifetime suicide planning and attempts could play an important role in interpreting the associations explored here. Additionally, the question employed to address suicidal “planning” (i.e., “Have you planned to complete suicide?”) may have been perceived by some participants as inquiring about one’s intention to suicide without necessarily involving preparatory steps. Therefore, future studies may seek to clarify such potentially confounding results. Fifthly, the current study specifically focused on the associations between psychological wellbeing, WSI and social support on suicidal behaviors; however, many other protective (e.g., coping strategies, or access to appropriate mental healthcare) and risk factors (e.g., income, alcohol and drugs, or previously diagnosed mental illness) influencing suicidal thoughts and behavior could be considered to provide a more systematic and more holistic approach to suicidal behaviors. For example, a prospective network analysis approach may enable simultaneously and comparatively assessing the effect of multiple such variables on suicidal behaviors [30]. Sixthly, the results reported here should be interpreted with caution, considering that suicide ideation was treated as a latent variable (i.e., composite score of three related questions) and limited empirical validity supports this methodological approach. Finally, the unexplored (in the present study) possibility of WSI operating as a moderator of the moderating influence of social support may also need to be explored in future research.

## Supplementary Information

**Additional file 1: Supplementary Table 1.** Demographic statistics. **Supplementary Table 2.** Reliability Analysis. **Supplementary Table 3.** Exploratory Factor Analysis. **Supplementary Table 4.** Johnson and Neyman technique to determine the zone of significance.

## Data Availability

The data that supports the findings of this study are available for Entertainment Assist, but restrictions apply to the availability of these data, which were used under license for the current study, and so are not publicly available. Data are however available for the authors upon reasonable request and with permission of Entertainment Assist. Please contact Daniel Zarate (daniel.zarate@live.vu.edu.au) to request access to the dataset.
