# Supplementary material for: Suicidal behaviors in the entertainment industry: a preliminary exploration of the interplay between work scheduling, social support, and wellbeing in Australia
Source: BMC Psychiatry. 2022 Dec 1;22:754. doi: 10.1186/s12888-022-04376-2 (PMC9714765; doi:10.1186/s12888-022-04376-2)
Supplement: Supplementary file 1 — Additional file 1: Supplementary Table 1. Demographic statistics. Supplementary Table 2. Reliability Analysis. Supplementary Table 3. Exploratory Factor Analysis. Supplementary Table 4. Johnson and Neyman technique to determine the zone of significance. [file 12888_2022_4376_MOESM1_ESM.docx]

|  | Supplementary Table 1. Demographic statistics | | | | | | | | | | | | |
| --- | --- | --- | --- | --- | --- | --- | --- | --- | --- | --- | --- | --- | --- |
|  | | N | MSPSS Family | MSPSS Friends | MSPSS Sig Other | MSPSS Total | SF12-MH | WSI | Suic Id 12 months | Suic Id Lifetime | Suicide Planning | Suicide Attempt |  |
|  | |  | Mean (*SD*) | Mean (*SD*) | Mean (*SD*) | Mean (*SD*) | Mean (*SD*) | Mean (*SD*) | Mean (*SD*) | Mean (*SD*) | Mean (*SD*) | Mean (*SD*) |  |
|  | |  |  |  |  |  |  |  |  |  |  |  |  |
| Age  18 – 24  25 – 40  40 – 65  65 + | | 203  605  484  10 | 3.64 (*1.06*)  3.55 (*1.04*)  3.36 (*1.10*)  3.35 (1*.20*) | 3.37 (*.97*)  3.36 (*.95*)  3.16 (*.93*)  3.60 (*.93*) | 3.70 (*1.13*)  3.90 (*1.16*)  3.69 (*1.15*)  4.02 (*.82*) | 3.57 (*.81*)  3.60 (*.80*)  3.41 (*.84*)  3.65 (*.82*) | 35.25 (*10.52*)  36.23 (*10.30*)  38.68 (*10.85*)  44.98 (*9.78*) | 3.38 (*.78*)  3.52 (*.82*)  3.46 (*.84*)  2.72 (*.87*) | 1.20 (*1.32*)  1.08 (*1.30*)  .94 (*1.25*)  .90 (*1.29*) | 1.98 (*1.24*)  1.97 (*1.24*)  1.83 (1*.26*)  1.60 (1*.35*) | .33 (*.47*)  .29 (*.46*)  .46 (*.85*)  .20 (*.42*) | .13 (*.33*)  .13 (*.34*)  .14 (*.35*)  .00 (*.00*) |  |
| Gender  Males  Females  Other / Non-binary | | 641  656  5 | 3.52 (*1.04*)  3.47 (*1.10*)  3.25 (*1.41*) | 3.34 (*.93*)  3.25 (*.96*)  3.30 (*1.04*) | 3.72 (*1.19*)  3.85 (*1.12*)  4.60 (*.55*) | 3.53 (*.84*)  3.52 (*.80*)  3.72 (*.87*) | 37.82 (*11.12*)  36.37 (*10.07*)  28.79 (*12.07*) | 3.46 (*.88*)  3.49 (*.77*)  3.40 (*.65*) | 1.00 (*1.29*)  1.09 (*1.28*)  1.40 (*1.52*) | 1.87 (*1.25*)  1.96 (*1.23*)  2.40 (*1.34*) | .31 (*.46*)  .30 (.*46*)  .40 (*.55*) | .13 (*.34*)  .14 (*.34*)  .20 *(.45*) |  |
|  | |  |  |  |  |  |  |  |  |  |  |  |  |
| Living arrangement  I live alone.  I live with someone. | | 213  1089 | 3.13 (*1.07*)  3.57 (*1.06*) | 3.13 (*.94*)  3.32 (.*94*) | 2.96 (*1.19*)  3.95 (*1.07*) | 3.07 (*.86*)  3.61 (*.78*) | 36.03 (*10.89*)  37.26 (*10.58*) | 3.49 (*.85*)  3.47 (*.82*) | 1.31 (*1.34*)  1.00 (*1.27*) | 2.07 (*1.18*)  1.89 (0*.85*) | .38 (*.49*)  .29 (*.45*) | .22 (*.41*)  .12 (.*32*) |  |
| Do you have a partner?  Yes  No | | 723  579 | 3.67 (*1.00*)  3.28 (*1.11*) | 3.31 (*.93*)  3.27 (*.97*) | 4.41 (*.71*)  3.02 (.*1.14*) | 3.79 (*.68*)  3.19 (*.85*) | 38.00 (*10.58*)  35.86 (*10.60*) | 3.46 (*.83*)  3.49 (*.82*) | .84 (1.21)  1.31 (*1.33*) | 1.79 (*1.28*)  2.07 (*1.19*) | .27 (.*45*)  .34 (*.47*) | .12 (*.32*)  .15 (*.36*) |  |
|  | |  |  |  |  |  |  |  |  |  |  |  |  |
| Do you have children?  Yes  No | | 399  903 | 3.59 (*1.05*)  3.45 (*1.08*) | 3.18 (*.93*)  3.34 (*.95*) | 3.97 (*1.03*)  3.71 (*1.20*) | 3.58 (*.80*)  3.50 (*.83*) | 39.26 (*10.66*)  36.08 (*10.49*) | 3.46 (*.86*)  3.48 (.*81*) | .82 (*1.20*)  1.15 (*1.31*) | 1.79 (*1.26*)  1.97 (1.24) | .29 (*.46*)  .31 (*.46*) | .13 (*.33*)  .14 (*.34*) |  |
| Highest education level  Up to year 12  Certif. / diploma  Bachelor  Post-grad. Degree  Total | | 286  375  431  210  1302 | 3.45 (*1.06*)  3.43 (*1.09*)  3.58 (*1.04*)  3.50 (*.1.10*)  3.49 (*1.07*) | 3.21 (*.96*)  3.31 (.*96*)  3.35 (.*95*)  3.25 (.*90*)  3.29 (.*95*) | 3.73 (*1.13*)  3.73 (*1.14*)  3.85 (*.1.16*)  3.83 (*1.19*)  3.79 (*.1.15*) | 3.46 (*.84*)  3.49 (.*82*)  3.60 (*.81*)  3.53 (.*80*)  3.52 (*.82*) | 36.91 (*11.00*)  37.35 (*.11.54*)  37.03 (*9.93*)  36.77 (*9.90*)  37.06 (*10.64*) | 3.56 (*.86*)  3.44 (*.83*)  3.45 (*.81*)  3.45 (*.80*)  3.47 (*.83*) | 1.20 (*1.34*)  1.11 (*1.32*)  .90 (*1.21*)  1.02 (*1.28*)  1.05 (*1.29*) | 1.91 (*1.24*)  1.93 (*1.24*)  1.85 (*1.26*)  2.03 (*1.22*)  1.92 (*1.25*) | .34 (*.47*)  .32 (*.47*)  .26 (.*44*)  .30 (.*46*)  .30 (.*46*) | .15 (*.36*)  .15 (*.36*)  .11 (*.31*)  .12 (*.32*)  .13 (*.34*) |  |
| *Note: MSPSS = Multidimensional scale of perceived social system; SF12-MH = Short form questionnaire mental health component; WSI = Work schedule impact; Suic Id = Suicidal ideation.* | | | | | | | | | | | | | |

**Supplementary table 2.** Reliability Analysis

| **MSPSS Overall**   \| Scale Reliability Statistics \| \| \| \| \| \| \| --- \| --- \| --- \| --- \| --- \| --- \| \|  \|  \|  \|  \|  \|  \| \|  \| \| **Cronbach's α** \| \| **McDonald's ω** \| \| \| scale \|  \| 0.906 \|  \| 0.907 \|  \| \|  \| \| \| \| \| \|      \| Item Reliability Statistics \| \| \| \| \| \| \| --- \| --- \| --- \| --- \| --- \| --- \| \|  \| \| **if item dropped** \| \| \| \| \|  \| \| **Cronbach's α** \| \| **McDonald's ω** \| \| \| MSPSS01 \|  \| 0.896 \|  \| 0.896 \|  \| \| MSPSS02 \|  \| 0.896 \|  \| 0.896 \|  \| \| MSPSS03 \|  \| 0.899 \|  \| 0.899 \|  \| \| MSPSS04 \|  \| 0.895 \|  \| 0.896 \|  \| \| MSPSS05 \|  \| 0.896 \|  \| 0.897 \|  \| \| MSPSS06 \|  \| 0.902 \|  \| 0.903 \|  \| \| MSPSS07 \|  \| 0.903 \|  \| 0.903 \|  \| \| MSPSS08 \|  \| 0.898 \|  \| 0.898 \|  \| \| MSPSS09 \|  \| 0.902 \|  \| 0.903 \|  \| \| MSPSS10 \|  \| 0.898 \|  \| 0.899 \|  \| \| MSPSS11 \|  \| 0.898 \|  \| 0.898 \|  \| \| MSPSS12 \|  \| 0.903 \|  \| 0.903 \|  \| \|  \| \| \| \| \| \| | **MSPSS Significant other**   \| Scale Reliability Statistics \| \| \| \| \| \| \| --- \| --- \| --- \| --- \| --- \| --- \| \|  \|  \|  \|  \|  \|  \| \|  \| \| **Cronbach's α** \| \| **McDonald's ω** \| \| \| scale \|  \| 0.953 \|  \| 0.953 \|  \| \|  \| \| \| \| \| \|      \| Item Reliability Statistics \| \| \| \| \| \| \| --- \| --- \| --- \| --- \| --- \| --- \| \|  \| \| **if item dropped** \| \| \| \| \|  \| \| **Cronbach's α** \| \| **McDonald's ω** \| \| \| MSPSS01 \|  \| 0.943 \|  \| 0.943 \|  \| \| MSPSS02 \|  \| 0.933 \|  \| 0.933 \|  \| \| MSPSS05 \|  \| 0.936 \|  \| 0.937 \|  \| \| MSPSS10 \|  \| 0.941 \|  \| 0.941 \|  \| \|  \| \| \| \| \| \| | **MSPSS Family**   \| Scale Reliability Statistics \| \| \| \| \| \| \| --- \| --- \| --- \| --- \| --- \| --- \| \|  \|  \|  \|  \|  \|  \| \|  \| \| **Cronbach's α** \| \| **McDonald's ω** \| \| \| scale \|  \| 0.922 \|  \| 0.923 \|  \| \|  \| \| \| \| \| \|      \| Item Reliability Statistics \| \| \| \| \| \| \| --- \| --- \| --- \| --- \| --- \| --- \| \|  \| \| **if item dropped** \| \| \| \| \|  \| \| **Cronbach's α** \| \| **McDonald's ω** \| \| \| MSPSS03 \|  \| 0.899 \|  \| 0.899 \|  \| \| MSPSS04 \|  \| 0.884 \|  \| 0.885 \|  \| \| MSPSS08 \|  \| 0.908 \|  \| 0.909 \|  \| \| MSPSS11 \|  \| 0.903 \|  \| 0.906 \|  \| \|  \| \| \| \| \| \| | **MSPSS Friends**   \| Scale Reliability Statistics \| \| \| \| \| \| \| --- \| --- \| --- \| --- \| --- \| --- \| \|  \|  \|  \|  \|  \|  \| \|  \| \| **Cronbach's α** \| \| **McDonald's ω** \| \| \| scale \|  \| 0.909 \|  \| 0.910 \|  \| \|  \| \| \| \| \| \|      \| Item Reliability Statistics \| \| \| \| \| \| \| --- \| --- \| --- \| --- \| --- \| --- \| \|  \| \| **if item dropped** \| \| \| \| \|  \| \| **Cronbach's α** \| \| **McDonald's ω** \| \| \| MSPSS06 \|  \| 0.880 \|  \| 0.882 \|  \| \| MSPSS07 \|  \| 0.882 \|  \| 0.883 \|  \| \| MSPSS09 \|  \| 0.886 \|  \| 0.889 \|  \| \| MSPSS12 \|  \| 0.882 \|  \| 0.884 \|  \| \|  \| \| \| \| \| \| |
| --- | --- | --- | --- | --- | --- | --- | --- | --- | --- | --- | --- | --- | --- | --- | --- | --- | --- | --- | --- | --- | --- | --- | --- | --- | --- | --- | --- | --- | --- | --- | --- | --- | --- | --- | --- | --- | --- | --- | --- | --- | --- | --- | --- | --- | --- | --- | --- | --- | --- | --- | --- | --- | --- | --- | --- | --- | --- | --- | --- | --- | --- | --- | --- | --- | --- | --- | --- | --- | --- | --- | --- | --- | --- | --- | --- | --- | --- | --- | --- | --- | --- | --- | --- | --- | --- | --- | --- | --- | --- | --- | --- | --- | --- | --- | --- | --- | --- | --- | --- | --- | --- | --- | --- | --- | --- | --- | --- | --- | --- | --- | --- | --- | --- | --- | --- | --- | --- | --- | --- | --- | --- | --- | --- | --- | --- | --- | --- | --- | --- | --- | --- | --- | --- | --- | --- | --- | --- | --- | --- | --- | --- | --- | --- | --- | --- | --- | --- | --- | --- | --- | --- | --- | --- | --- | --- | --- | --- | --- | --- | --- | --- | --- | --- | --- | --- | --- | --- | --- | --- | --- | --- | --- | --- | --- | --- | --- | --- | --- | --- | --- | --- | --- | --- | --- | --- | --- | --- | --- | --- | --- | --- | --- | --- | --- | --- | --- | --- | --- | --- | --- | --- | --- | --- | --- | --- | --- | --- | --- | --- | --- | --- | --- | --- | --- | --- | --- | --- | --- | --- | --- | --- | --- | --- | --- | --- | --- | --- | --- | --- | --- | --- | --- | --- | --- | --- | --- | --- | --- | --- | --- | --- | --- | --- | --- | --- | --- | --- | --- | --- | --- | --- | --- | --- | --- | --- | --- | --- | --- | --- | --- | --- | --- | --- | --- | --- | --- | --- | --- | --- | --- | --- | --- | --- | --- | --- | --- | --- | --- | --- | --- | --- | --- | --- | --- | --- | --- | --- | --- | --- | --- | --- | --- | --- | --- | --- | --- | --- | --- | --- | --- | --- | --- | --- | --- | --- | --- | --- | --- | --- | --- | --- | --- | --- | --- | --- | --- | --- | --- | --- | --- | --- | --- | --- | --- | --- | --- | --- | --- | --- | --- | --- | --- | --- | --- | --- | --- | --- | --- | --- | --- | --- | --- | --- | --- | --- | --- | --- | --- | --- | --- | --- | --- | --- | --- | --- | --- | --- | --- | --- | --- | --- | --- | --- |

**Supplementary table 2 (continued).** Reliability Analysis

| **Short form questionnaire - SF12**   \| Scale Reliability Statistics \| \| \| \| \| \| \| --- \| --- \| --- \| --- \| --- \| --- \| \|  \|  \|  \|  \|  \|  \| \|  \| \| **Cronbach's α** \| \| **McDonald's ω** \| \| \| scale \|  \| 0.876 \|  \| 0.878 \|  \| \|  \| \| \| \| \| \|      \| Item Reliability Statistics \| \| \| \| \| \| \| --- \| --- \| --- \| --- \| --- \| --- \| \|  \| \| **if item dropped** \| \| \| \| \|  \| \| **Cronbach's α** \| \| **McDonald's ω** \| \| \| SF12_1 \|  \| 0.866 \|  \| 0.868 \|  \| \| SF12_2 \|  \| 0.874 \|  \| 0.875 \|  \| \| SF12_3 \|  \| 0.874 \|  \| 0.875 \|  \| \| SF12_4 \|  \| 0.861 \|  \| 0.864 \|  \| \| SF12_5 \|  \| 0.862 \|  \| 0.865 \|  \| \| SF12_6 \|  \| 0.856 \|  \| 0.861 \|  \| \| SF12_7 \|  \| 0.862 \|  \| 0.866 \|  \| \| SF12_8 \|  \| 0.872 \|  \| 0.873 \|  \| \| SF12_9 \|  \| 0.868 \|  \| 0.872 \|  \| \| SF12_10 \|  \| 0.867 \|  \| 0.870 \|  \| \| SF12_11 \|  \| 0.864 \|  \| 0.868 \|  \| \| SF12_12 \|  \| 0.859 \|  \| 0.863 \|  \| \|  \| \| \| \| \| \| | \| **Work Scheduling impact - WSI**  Scale Reliability Statistics \| \| \| \| \| \| \| --- \| --- \| --- \| --- \| --- \| --- \| \|  \|  \|  \|  \|  \|  \| \|  \| \| **Cronbach's α** \| \| **McDonald's ω** \| \| \| scale \|  \| 0.805 \|  \| 0.809 \|  \| \|  \| \| \| \| \| \|      \| Item Reliability Statistics \| \| \| \| \| \| \| --- \| --- \| --- \| --- \| --- \| --- \| \|  \| \| **if item dropped** \| \| \| \| \|  \| \| **Cronbach's α** \| \| **McDonald's ω** \| \| \| WSI1 \|  \| 0.775 \|  \| 0.780 \|  \| \| WSI2 \|  \| 0.762 \|  \| 0.772 \|  \| \| WSI3 \|  \| 0.762 \|  \| 0.766 \|  \| \| WSI4 \|  \| 0.775 \|  \| 0.784 \|  \| \| WSI5 \|  \| 0.765 \|  \| 0.778 \|  \| \| WSI6 \|  \| 0.805 \|  \| 0.809 \|  \| \|  \| \| \| \| \| \| | **Suicidality questions**   \| Scale Reliability Statistics \| \| \| \| \| \| \| --- \| --- \| --- \| --- \| --- \| --- \| \|  \|  \|  \|  \|  \|  \| \|  \| \| **Cronbach's α** \| \| **McDonald's ω** \| \| \| scale \|  \| 0.865 \|  \| 0.865 \|  \| \|  \| \| \| \| \| \|      \| Item Reliability Statistics \| \| \| \| \| \| \| --- \| --- \| --- \| --- \| --- \| --- \| \|  \| \| **if item dropped** \| \| \| \| \|  \| \| **Cronbach's α** \| \| **McDonald's ω** \| \| \| Suic1 \|  \| 0.847 \|  \| 0.852 \|  \| \| Suic2 \|  \| 0.842 \|  \| 0.847 \|  \| \| Suic3 \|  \| 0.851 \|  \| 0.855 \|  \| \| Suic4 \|  \| 0.835 \|  \| 0.836 \|  \| \| Suic5 \|  \| 0.836 \|  \| 0.837 \|  \| \| Suic6 \|  \| 0.840 \|  \| 0.841 \|  \| \|  \| \| \| \| \| \| |
| --- | --- | --- | --- | --- | --- | --- | --- | --- | --- | --- | --- | --- | --- | --- | --- | --- | --- | --- | --- | --- | --- | --- | --- | --- | --- | --- | --- | --- | --- | --- | --- | --- | --- | --- | --- | --- | --- | --- | --- | --- | --- | --- | --- | --- | --- | --- | --- | --- | --- | --- | --- | --- | --- | --- | --- | --- | --- | --- | --- | --- | --- | --- | --- | --- | --- | --- | --- | --- | --- | --- | --- | --- | --- | --- | --- | --- | --- | --- | --- | --- | --- | --- | --- | --- | --- | --- | --- | --- | --- | --- | --- | --- | --- | --- | --- | --- | --- | --- | --- | --- | --- | --- | --- | --- | --- | --- | --- | --- | --- | --- | --- | --- | --- | --- | --- | --- | --- | --- | --- | --- | --- | --- | --- | --- | --- | --- | --- | --- | --- | --- | --- | --- | --- | --- | --- | --- | --- | --- | --- | --- | --- | --- | --- | --- | --- | --- | --- | --- | --- | --- | --- | --- | --- | --- | --- | --- | --- | --- | --- | --- | --- | --- | --- | --- | --- | --- | --- | --- | --- | --- | --- | --- | --- | --- | --- | --- | --- | --- | --- | --- | --- | --- | --- | --- | --- | --- | --- | --- | --- | --- | --- | --- | --- | --- | --- | --- | --- | --- | --- | --- | --- | --- | --- | --- | --- | --- | --- | --- | --- | --- | --- | --- | --- | --- | --- | --- | --- | --- | --- | --- | --- | --- | --- | --- | --- | --- | --- | --- | --- | --- | --- | --- | --- | --- | --- | --- | --- | --- | --- | --- | --- | --- | --- | --- | --- | --- | --- | --- | --- | --- | --- | --- | --- | --- | --- | --- | --- | --- | --- | --- | --- | --- | --- | --- | --- | --- | --- | --- | --- | --- | --- | --- | --- | --- | --- | --- | --- | --- | --- | --- | --- | --- | --- | --- | --- | --- | --- | --- | --- | --- | --- | --- | --- | --- | --- | --- | --- | --- | --- | --- | --- | --- | --- | --- | --- | --- | --- | --- |

**Supplementary table 3.** Exploratory Factor Analysis

| \| \| Factor Loadings \| \| \| \| \| \| \| \| \| --- \| --- \| --- \| --- \| --- \| --- \| --- \| --- \| \|  \| \| **Factor** \| \| \| \|  \| \| \|  \| \| **1** \| \| **2** \| \| **Uniqueness** \| \| \| WSI1 \|  \| 0.796 \|  \|  \|  \| 0.378 \|  \| \| WSI2 \|  \| 0.896 \|  \|  \|  \| 0.222 \|  \| \| WSI3 \|  \| 0.547 \|  \|  \|  \| 0.510 \|  \| \| WSI4 \|  \|  \|  \| 0.667 \|  \| 0.492 \|  \| \| WSI5 \|  \|  \|  \| 0.921 \|  \| 0.183 \|  \| \| WSI6 \|  \|  \|  \| 0.495 \|  \| 0.727 \|  \| \| Note. 'Minimum residual' extraction method was used in combination with a 'oblimin' rotation \| \| \| \| \| \| \| \| \|  \| \| \| \| \| \| \| \| \| \| --- \| --- \| --- \| --- \| --- \| --- \| --- \| --- \| --- \| --- \| --- \| --- \| --- \| --- \| --- \| --- \| --- \| --- \| --- \| --- \| --- \| --- \| --- \| --- \| --- \| --- \| --- \| --- \| --- \| --- \| --- \| --- \| --- \| --- \| --- \| --- \| --- \| --- \| --- \| --- \| --- \| --- \| --- \| --- \| --- \| --- \| --- \| --- \| --- \| --- \| --- \| --- \| --- \| --- \| --- \| --- \| --- \| --- \| --- \| --- \| --- \| --- \| --- \| --- \| --- \| --- \| --- \| --- \| --- \| --- \| --- \| --- \| --- \| --- \| --- \| --- \| --- \| --- \| --- \| --- \| --- \| --- \| --- \| --- \| --- \| --- \| --- \| --- \| --- \| | \| KMO Measure of Sampling Adequacy \| \| \| \| \| --- \| --- \| --- \| --- \| \|  \|  \|  \|  \| \|  \| \| **MSA** \| \| \| Overall \|  \| 0.763 \|  \| \| WSI1 \|  \| 0.739 \|  \| \| WSI2 \|  \| 0.727 \|  \| \| WSI3 \|  \| 0.855 \|  \| \| WSI4 \|  \| 0.751 \|  \| \| WSI5 \|  \| 0.729 \|  \| \| WSI6 \|  \| 0.815 \|  \| \|  \| \| \| \| | \| Bartlett's Test of Sphericity \| \| \| \| \| \| \| --- \| --- \| --- \| --- \| --- \| --- \| \|  \|  \|  \|  \|  \|  \| \| **χ²** \| \| **df** \| \| **p** \| \| \| 2861 \|  \| 15 \|  \| < .001 \|  \| \|  \| \| \| \| \| \| |
| --- | --- | --- | --- | --- | --- | --- | --- | --- | --- | --- | --- | --- | --- | --- | --- | --- | --- | --- | --- | --- | --- | --- | --- | --- | --- | --- | --- | --- | --- | --- | --- | --- | --- | --- | --- | --- | --- | --- | --- | --- | --- | --- | --- | --- | --- | --- | --- | --- | --- | --- | --- | --- | --- | --- | --- | --- | --- | --- | --- | --- | --- | --- | --- | --- | --- | --- | --- | --- | --- | --- | --- | --- | --- | --- | --- | --- | --- | --- | --- | --- | --- | --- | --- | --- | --- | --- | --- | --- | --- | --- | --- | --- | --- | --- | --- | --- | --- | --- | --- | --- | --- | --- | --- | --- | --- | --- | --- | --- | --- | --- | --- | --- | --- | --- | --- | --- | --- | --- | --- | --- | --- | --- | --- | --- | --- | --- | --- | --- | --- | --- | --- | --- | --- | --- | --- | --- | --- | --- | --- | --- | --- | --- | --- | --- | --- | --- | --- | --- | --- | --- | --- | --- | --- | --- | --- | --- | --- | --- | --- | --- | --- | --- | --- | --- | --- |

**Supplementary table 4.** Johnson and Neyman technique to determine the zone of significance.

Moderator value(s) defining Johnson-Neyman significance region(s):

Value % below % above

9.7816 84.6390 15.3610

Conditional X*W interaction at values of the moderator Z:

MSPSS_T Effect se t p LLCI ULCI

-30.2995 .0033 .0017 1.9436 .0522 .0000 .0067

-27.8995 .0030 .0016 1.9027 .0573 -.0001 .0062

-25.4995 .0028 .0015 1.8542 .0639 -.0002 .0057

-23.0995 .0025 .0014 1.7962 .0727 -.0002 .0052

-20.6995 .0022 .0013 1.7260 .0846 -.0003 .0047

-18.2995 .0019 .0012 1.6398 .1013 -.0004 .0042

-15.8995 .0016 .0011 1.5326 .1256 -.0005 .0038

-13.4995 .0014 .0010 1.3977 .1624 -.0006 .0033

-11.0995 .0011 .0009 1.2259 .2205 -.0007 .0028

-8.6995 .0008 .0008 1.0055 .3148 -.0008 .0024

-6.2995 .0005 .0007 .7235 .4695 -.0009 .0019

-3.8995 .0002 .0007 .3690 .7122 -.0011 .0015

-1.4995 .0000 .0006 -.0580 .9538 -.0012 .0012

.9005 -.0003 .0006 -.5352 .5926 -.0015 .0008

3.3005 -.0006 .0006 -1.0147 .3105 -.0017 .0006

5.7005 -.0009 .0006 -1.4412 .1498 -.0021 .0003

8.1005 -.0012 .0006 -1.7800 .0753 -.0024 .0001

9.7816 -.0014 .0007 -1.9618 .0500 -.0027 .0000

10.5005 -.0014 .0007 -2.0268 .0429 -.0028 .0000

12.9005 -.0017 .0008 -2.1970 .0282 -.0033 -.0002

15.3005 -.0020 .0009 -2.3110 .0210 -.0037 -.0003

17.7005 -.0023 .0010 -2.3862 .0172 -.0042 -.0004
